# Citrullination: new tricks for an old mod

**DOI:** 10.1098/rstb.2022.0235

**Published:** 2023-11-20

**Authors:** Maria A. Christophorou, Priyanka Sharma, Xuesen Zhang

**Affiliations:** ^1^ Babraham Institute, Cambridge CB22 3AT, UK; ^2^ Institute of Pharmacology and Structural Biology (IPBS-UMR5089), 205 Rte de Narbonne, Toulouse 31400, France; ^3^ College of Basic Medical Science, China Medical University, Shenyang 110122, China

## 1. Introduction

Proteins are subject to a myriad of chemical modifications that modulate their structure, stability, function and interaction with other molecules, adding an enormous amount of complexity and scope for regulation to biological systems. Such post-translational modifications (PTMs) can be elicited by cellular stimuli or stresses and initiate downstream responses, making cells adaptable to their environment and mediating changes such as proliferation, differentiation and death. Citrulline can be present within proteins as a result of a post-translational modification of arginine residues, called peptylarginine deimination, or citrullination. As citrulline is a non-coded amino acid, its presence within a protein signifies a stimulus and a response. Although citrullination was first demonstrated in the 1960s [1] and the first of the citrullinating enzymes, the peptidylarginine deiminases (PADIs or PADs), was isolated in the early 1980s [2], there is still an ever-increasing number of cellular activities and pathologies shown to be impacted by it, with considerable advances made in the last 15–20 years. It is now understood that the small family of five PADI enzymes has many physiological and pathophysiological functions (reviewed in [3]), however, we still lack fundamental understanding of the mechanistic principles of PADI regulation within cells and the mechanisms through which they carry out their cellular and organismal functions.

Our understanding of citrullination stems from many and diverse fields including neurobiology, immunology, reproductive biology, skin physiology, cell signalling, chromatin biology and transcription, as well as autoimmunity, neurodegeneration and cancer. Although the regulatory scope of PADIs is clearly wide, these enzymes exhibit high sequence and structural conservation, suggesting that certain mechanistic principles are likely to apply to the regulation of different isozymes. Additionally, the impressive recent advances in analytical methodologies, such as targeted mass spectrometry and chemical biology efforts to modulate PADI function, may be applied to many different biological systems. It therefore became obvious that a forum was needed for scientists from the different facets of citrullination research to come together, discuss their work and exchange ideas, in order to catalyse progress in the field. The first international conference on protein citrullination therefore took place in October 2022 in the United Kingdom, with generous support from the Royal Society (https://royalsociety.org/science-events-and-lectures/2022/10/protein-citrullination/). This discussion meeting brought together scientists focusing on cell and developmental biology, cell signalling, gene transcription, cancer biology and autoimmunity, while it incorporated key presentations from leading experts in mass spectrometry and pharmacology. The current theme issue follows this meeting to report recent work from the participants and includes nine research papers and six review articles, which cover a wide variety of topics. In this Introduction we summarize the advances presented in the issue, which include both new mechanistic understanding of established PADI functions and emerging new themes in citrullination biology.

## 2. In this issue—new functions and new perspectives on established ones

### (a) Cell differentiation and tissue biology

Beginning with the two oldest known tissues to harbour citrullination, the skin and the brain, we have a comprehensive review of roles of PADIs in skin physiology and inflammatory disease by Méchin & Simon [4], while Genander and colleagues add a new perspective on *Padi* gene regulation, with the report of new *Padi2* and *Padi3* isoforms in fair follicle and oligodendrocyte progenitor cells, respectively (Patil *et al.* [5]). This study reports that in contrast to the known function of canonical PADI2 [6]*,* the newly identified PADI2b inhibits oligodendrocyte differentiation. Additionally, the authors identify the isoform PADI3b and suggest that it may counteract the activity of canonical PADI3 in skin differentiation by reducing its stability and enzymatic activity. These findings suggest that PADI regulation is significantly more complex than appreciated previously and provide new mechanistic understanding of cell differentiation and tissue development.

Expanding on our understanding of the roles of PADIs in cell differentiation, a study by the Christophorou laboratory probes the role of PADI4 in the regulation of multi-lineage differentiation potential of embryonic stem cells (Singh *et al.* [7]). PADI4 has been implicated in the regulation of pluripotency, in embryonic stem (ES) and induced pluripotent stem cells [8,9]. Here, through a series of complementary bulk and single-cell transcriptomic analyses, the authors find that *Padi4* deletion impairs the potential of ES cells to produce mesoderm, cardiomyocytes and endothelial cells in a model of embryoid body differentiation, suggesting that PADI4 may mediate differentiation of the mesodermal lineage during embryonic development and implicating it in cardiac development for the first time. This is particularly intriguing in light of work by the Martinod laboratory. Here, the authors expand on their previous findings that PADI4 is necessary for tissue fibrosis associated with cardiac ageing [10], by demonstrating that the source of fibrosis-promoting PADI4 is circulating neutrophils (Van Bruggen *et al.* [11]). Together, these two studies suggest that PADI4 has important roles in cardiac tissue biology, with opposing effects in development and ageing—during development, cell-autonomous PADI4-mediated transcriptional regulation may promote heart differentiation, while during ageing, non-cell-autonomous citrullination stemming for neutrophil PADI4 has detrimental effects on the same tissue. Considering the emerging roles of PADIs in cell differentiation and their well-established roles in inflammatory disease, it will be interesting to understand whether this dichotomy is a general feature of PADI biology in animals.

### (b) Citrullination and the extracellular matrix

A prominent theme in this issue explores the role of citrullination in the regulation of the extracellular matrix (ECM). The two studies mentioned above, which suggest a role for PADI4 in heart development and demonstrate a non-cell-autonomous role in heart fibrosis, both report that loss of PADI4 leads to the reduction of genes associated with the ECM (Singh *et al.* [7]*;* Van Bruggen *et al.* [11]). Additionally, an excellent review by Saifi & Ho, details the current knowledge on the mechanisms and functions associated with citrullination of matrisomal proteins [12]. The authors focus in particular on how aberrant citrullination of such proteins may impact disease development and the clinical potential of manipulating ECM protein citrullination. The study of how PADIs affect the composition (through regulation of gene expression) and properties (through enzymatic modification) of ECM proteins promises to be a fertile research area, with important implications in therapies against ageing and disease, not least because it may allow for the manipulation of cells through the extracellular environment, obviating the need for genetic manipulation.

### (c) Peptidylarginine deiminases in unexpected places

Further on the potential of PADIs to reach beyond their classically understood cellular environment, an intriguing study by the Darrah laboratory reports that PADI4, which harbours a nuclear localization signal and is known to localize predominantly to the nucleus, is found to be associated with cellular organelles of human monocytes from healthy donors (Thomas *et al.* [13]). While the presence of PADI4 on the cell surface of monocytes was demonstrated previously [14], and indeed suggested by the fact that extracellular proteins are citrullinated as described above, this systematic study suggests that PADI4 associates with the vesicular organelle fraction of monocytes, presents *in vitro* evidence that it can bind organelle proteins and identifies organelle-specific PADI4 substrates after monocyte stimulation and PADI4 activation. In light of these findings, it is interesting to consider data presented by Singh *et al.* [7]*,* which show that PADI4 loss in ES cells leads to perturbation in the expression of genes associated with vesicular organelles. Collectively, these findings may have important implications regarding PADI4-regulated cellular functions under normal and pathological conditions. It will be particularly exciting to understand whether the extra-nuclear localization of PADI4 is a particular feature of monocytes or whether it also applies to other cell types such, as pluripotent stem cells and cancer cells.

### (d) Innate immunity and autoimmunity

One of the best-studied aspects of PADI function is in neutrophil responses to infection and in sterile inflammation. A substantial body of literature probes the role of PADIs in the formation of neutrophil extracellular traps (NETs) [15] and their role in innate immunity, autoimmunity, atheroschlerosis and tissue inflammation (reviewed in [16]). Two studies within this issue probe the much-debated role of citrullination in NET formation, function and autoreactivity [16]. Bont & Pruijn compiled proteomic data from previous studies that assessed the presence of autoantibodies in autoimmune disease and suggest that citrulline-containing epitopes do not account for the majority of NET-specific autoantibodies from patients with rheumatoid arthritis (RA) and systemic lupus erythematosus (SLE) [17]. They support this suggestion by testing sera from RA and SLE patients for reactivity against NETs produced with different stimuli. This work is intriguing, considering the fact that anti-citrullinated protein autoantibodies [18] consist a key diagnostic and prognostic tool for RA in clinical practice, and may warrant a more careful look at refining the use of such biomarkers to increase the sensitivity of diagnostic tests. A second study by Martinod and colleagues probes role of PADI4 and PADI2 in NETs and the regulation of von Willebrand factor and ADAMTS13 (a disintegrin and metalloproteinase with thrombospondin type 1 motifs, member 13) in the innate immune response to bacterial infection, using bacteremia patient samples (Martens *et al.* [19]). Increased understanding of the neutrophil response to bacterial infection may allow improved methods of combating bacteremia in patients while preventing the deleterious consequences of sepsis. Additionally, a study by Neeli & Radic suggests a new mechanism of PADI4 activation through integrin-mediated neutrophil attachment and provides supporting evidence for a role of citrullination in neutrophil chemotaxis [20]. The notion that PADI4 is involved in neutrophil chemotaxis is also supported by data presented by Van Bruggen *et al.* [11]*,* where it is reported that chemotactic gene expression is reduced in the hearts of mice deficient for neutrophil PADI4 [11], although the mechanism through which this is mediated remains to be determined.

### (e) Cancer

Much of our knowledge of how citrullination impacts cell signalling and gene regulation comes from cancer biology studies. Aberrant PADI expression and activity are features of a variety of malignant tumours and separate them from their benign or normal tissue counterparts [21]. Consequently, cancer cell lines have traditionally provided useful systems for studying PADI-mediated signaling and transcription. A comprehensive review article by the La Thangue laboratory summarizes the current knowledge of citrullination in cancer and details known mechanisms of cross-talk between citrullination and other PTMs (Koyo *et al.* [22]). This article provides an excellent primer for future research that will both help understand mechanisms of action of PADIs in pathophysiology and help integrate citrullination into better-understood molecular mechanisms and cellular functions. In addition, a research article by the Zhang laboratory builds on their previously identified mechanism of PADI2-mediated MEK1 citrullination and downstream signalling in endometrial cancer [23]. Here, the authors assess the potential for interfering with this mechanism as a cancer therapy in multiple tumours (Xue *et al.* [24]). They show that inhibition of MEK1 citrullination enhances the effects of the chemotherapeutic agent docetaxel in inhibiting cancer stem cell maintenance. Importantly, the authors show that PADI2 inhibition restores chemosensitivity in resistant cancer cells. The incorporation of PADI modulators into cancer therapy promises to increase its efficacy for a number of blood and solid tumours. Mechanistic understanding of the roles of PADI deregulation in cancer will be essential in designing rational therapies.

### (f) PADI6, the rogue member of the family

While the members of the mammalian *Padi* genes arose from gene duplication and share structural similarity, one member stands out. PADI6 is less conserved, is missing key calcium binding residues that are necessary for catalysis, suggesting it has no catalytic activity, and has no reported substrates. Additionally, PADI6 expression is highly specific to oocytes and early embryos and is down-regulated early in embryonic development. As a result, PADI6 is studied much less frequently. However, PADI6 has key roles in oocyte maturation and early embryonic development, with its loss leading to female infertility and early embryo arrest. Williams & Walport [25] contribute a comprehensive review on PADI6. In our quest to understand citrullination, it is important to consider the functions of PADI6 in the oocyte—while this is a highly specialized cell with unique biology, it is plausible that the non-catalytic functions of PADI6 are mirrored by other PADIs in different contexts.

### (g) Detection, modulation and incorporation of citrullines

The development of analytical technologies for the detection of citrullination has been crucial for the progress observed in the field in the last two decades. A considerable array of methods exist for the proteomic and chemical detection of citrullination. Two review articles in this issue, written by the Nielsen and Thompson laboratories, summarize the state of the art of citrullination detection and discuss the challenges associated with the different methodologies (Rebak *et al.* [26]; Barasa & Thompson [27]). These articles consist a valuable resource for researchers interested in understanding citrullination, or in developing new and improved analytical methods. Additionally, the review by Barasa & Thompson discusses an important approach developed by the laboratory [28], for incorporating citrulline residues within proteins in mammalian cells. This approach will be necessary in accurately attributing biological functions to specific citrullination events, something that constitutes a significant challenge when studying enzymes with no apparent consensus target sequence, such as the PADIs.

Another key facilitating factor in the study of citrullination has been the development of PADI inhibitors. In their article, Barasa & Thompson discuss the currently available isozyme-specific PADI inhibitors and their properties. Additionally, a study by Ke and colleagues reports a naturally occurring PADI2 activating molecule, demethoxycurcumin (Zhang *et al.* [29]). Along with nitazoxanide, which was previously described by the same team [30], this compound may be of significant value in cancer biology contexts where PADI2 is deregulated [30]. Additionally, PADI-activating compounds are likely to become highly valuable reagents towards our understanding of the cellular functions impacted by PADIs.

## 3. Concluding remarks

A disproportionally small amount is known about the regulation of cellular PADIs. This is in stark contrast with the number of pathophysiological and, increasingly, physiological functions that have been attributed to these enzymes. Additionally, much remains to be understood regarding how pathological levels of citrullination arise in different disease contexts, compared to physiological levels. Deeper understanding of how PADI physiology differs from pathophysiology may offer a crucial window for therapeutic intervention. The advances described in this theme issue and the increasing efforts for dialogue and collaboration within the field stand to generate important new knowledge in the areas of tissue biology, cellular trafficking, epigenetics and signal transduction and lead to improved new therapies against a variety of pathologies. There has never been a more exciting time to study citrullination and PADI enzymes and we encourage scientists who come across them in their research to contact the authors represented in here for discussion and collaboration.

## Data Availability

This article has no additional data.
